# Thermodynamic Properties of Some Methylphosphonyl Dihalides From 15 to 335 °K^1^

**DOI:** 10.6028/jres.068A.036

**Published:** 1964-08-01

**Authors:** George T. Furukawa, Martin L. Reilly, Jeanette H. Piccirelli, Milton Tenenbaum

## Abstract

Measurements of the heat capacity of methylphosphonyl difluoride (CH_3_POF_2_), methyl phosphonyl dichloride (CH_3_POCl_2_), and methylphosphonyl chlorofluoride (CH_3_POClF) were made from about 15 to 335 °K by means of an adiabatic calorimeter. These highly reactive and toxic substances were purified in a completely closed glass apparatus by combining slow crystallization and fractional melting procedures. The purities determined by the freezing-curve method are shown to be generally in agreement with those values obtained by the calorimetric method. From the results of the heat measurements, the triple-point temperature, heat of fusion, and their corresponding estimated uncertainties were found to be, respectively, 236.34±0.05 °K and 11,878±12 J/mole for CH_3_POF_2_, 306.14± 0.02 °K and 18,076±15 J/mole for CH_3_POCl_2_, and 250.70± 0.20 °K and 11,853±30 J/mole for CH_3_POClF. Triple-point temperatures obtained by the freezing-curve method are in agreement with the above values. A table of smoothed values of heat capacity, enthalpy, enthalpy function, entropy, Gibbs free energy, and Gibbs free energy function from 0 to 335 °K was obtained from the data. The entropy and its corresponding estimated uncertainty for CH3POF2, CH3POCl2, and CH3POClF in their respective condensed phase at 298.15 °K and saturation pressure was found to be 208.3± 0.3, 164.8± 0.3, and 216.4± 0.4 J/deg mole, respectively. The entropies in the gaseous state at 298.15 °K and 1 atm pressure were found to be 312.7±3, 339.7±3, and 335.0±3 J/deg mole, respectively.

## 1. Introduction

This paper describes the method of purification and the results of heat-capacity measurements obtained on methylphosphonyl difluoride (CH_3_POF_2_), methylphosphonyl dichloride (CH_3_POCl_2_), and methylphosphonyl chlorofluoride (CH_3_POClF) from about 15 to 335°K. (Henceforth, these compounds will be referred to synonymously as difluoro, dichloro, and chlorofluoro, respectively.) The purity and the triple-point temperature were determined by both freezing-curve and calorimetric melting-curve methods and the results compared. Heats of fusion, triple-point temperatures, and tables of smoothed values of heat capacity, enthalpy, enthalpy function, entropy, Gibbs free energy, and Gibbs free energy function were calculated from the calorimetric data. The entropies of these substances in the gas phase at 1 atm pressure and 298.15 °K were calculated using the adjuvant data given in the literature.

Phosphorus compounds are being used in an increasing variety of purposes—pharmaceuticals, special lubricants, insecticides, and others. Accurate thermodynamic data on CH_3_POF_2_, CH_3_POCl_2_, and CH_3_POClF are expected to be of considerable technological interest.

## 2. Calorimetric Apparatus and Method

Measurements of the heat capacity were made in an adiabatic calorimeter of a design similar to that previously described [ 10].^3^ Briefly, the sample vessel of about 50-ml capacity was constructed of platinum (90%)—iridium (10%) alloy with pure platinum vanes arranged radially in a manner similar to the copper vessel shown in reference [10]. The vanes were spot-welded at numerous places to the vessel wall and to the central reentrant well wall. The reentrant well was constructed to match as closely as possible the outer casing of a removable heater-platinum resistance thermometer assembly. To improve the thermal contact a thin layer of stopcock grease (Apiezon T) was applied to the outer casing of the heater-thermometer assembly before inserting into the reentrant well. The amount of grease used was accurately known and was maintained the same in the heat measurements on both the vessel-plus-sample and on the empty-vessel experiments. A thin gold-plated copper shell (0.015 in. thick) enclosed the container so that a surface more reproducible [8] in temperature than that of the platinum-iridium vessel could be provided for adiabatic control. The vessel and the thin copper shell were in good thermal contact around the girth of the vessel. The heater-thermometer leads were also in good thermal contact with the thin copper shell.

The calorimeter vessel with its heater-thermometer assembly and gold-plated copper shell was suspended from a thin linen cord within the adiabatic shield system. The inner surface of the adiabatic shield was also gold plated to minimize the heat transfer through radiation. The space surrounding the vessel and adiabatic shield was evacuated to a pressure of 10^−5^ torr (1 torr= 1/760 atm=l mm Hg) or less to make negligible the heat transfer by gaseous conduction and convection. The adiabatic shield temperature was controlled by manual adjustment of the current through the shield heaters in conjunction with constantan-Chromel-P differential thermocouples.^4^

The electrical power input was determined by means of a Wenner potentiometer in conjunction with a standard cell, volt box, and standard resistor. The time interval of heating was measured by means of a precision timer operated on 60 Hz frequency-standard furnished by the Electrical Instruments Section of the National Bureau of Standards. The stability of the frequency is better than 0.5 ppm. Temperatures were measured by means of the platinum-resistance thermometer and a high-precision Mueller bridge. The platinum-resistance thermometer was calibrated above 90 °K in accordance with the 1948 International Practical Temperature Scale [12, 13], and between 10 and 90 °K on the NBS–1955 provisional temperature scale. The provisional temperature scale as it is presently maintained at the National Bureau of Standards, and referred to as degrees K (NBS–1955), is, by definition, 0.01 deg lower than the former NBS–1939 scale [6]. The temperatures in degrees Kelvin above 90 °K were obtained by adding 273.15 deg to the temperatures in degrees Celsius (International Practical Temperature Scale of 1948 [13]), in accordance with the definition of the thermodynamic temperature scale by assigning 273.16 °K to the triplepoint of water [13].

The molecular weights used were obtained from the table of atomic weights based on carbon 12 adopted in 1961 [7].

## 3. Preparation of the Samples

The extremely toxic and reactive nature of these substances dictated the purification and subsequent handling procedures to be performed in a closed glass system. For the purification, the fractional crystallization and melting method was selected for its convenience in handling the substances in vacuum and no temperature higher than the melting points of the compounds being required. An increase in the rate of disproportionation of chlorofluoro to difluoro and dichloro at the higher temperatures was also considered very likely. Visual observations indicated that the dichloro compound (mp=30 °C) tended to grow in rather large crystals and might be susceptible to purification by fractional crystallization and melting procedures.

Previous investigations on purification by the fractional crystallization and melting process reported from the NBS [1] indicated that with some substances a major portion (about ¾) of the impurities is removed along with the first 20-percent fraction melted. The following successive 20-percent fraction melted removed about of the remaining impurities. A preliminary determination of the purity of the difluoro sample before purification gave 99.7 mole percent. With a starting material of this purity, the last 40-percent fraction melted was expected to be 99.95 mole percent pure if the efficiency reported in reference [1] were achieved.

The apparatus used in the purification process has been described previously [1]. The details of slow crystallization and melting procedures used with the completely closed apparatus are given in the reference cited. The various cooling and warming baths used in the crystallization and melting processes are summarized in columns 5 and 6 of table 1, respectively. The last 40 percent to melt was collected in a single receiver and used subsequently for the determination of purity by the freezing-curve method and for the calorimetric measurements.

## 4. Determination of Purity and Triple-Point Temperatures

### 4.1. Freezing-Curve Method

The procedures for determining the purity and triple-point temperature from time-temperature freezing curves and the principles involved have previously been described [2, 3, 4].

The apparatus used for the determination of purity by the freezing-curve method was designed especially for reactive substances that must be contained within a closed system. The details of the apparatus and the procedures for handling samples and making measurements in the apparatus have been given previously [4].

**Figure 1:**
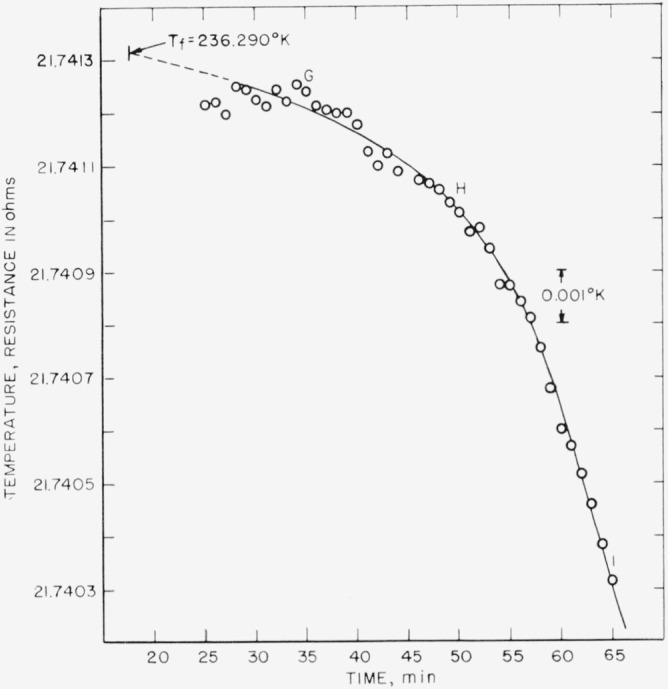
Freezing curve of methylphosphonyl difluoride at saturation pressure.

**Figure 2:**
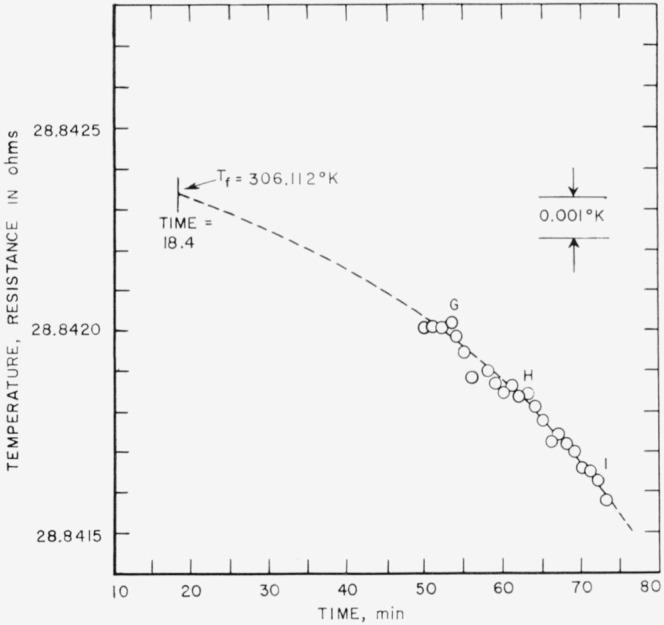
Freezing curve of methylphosphonyl dichloride at saturation pressure.

**Figure 3:**
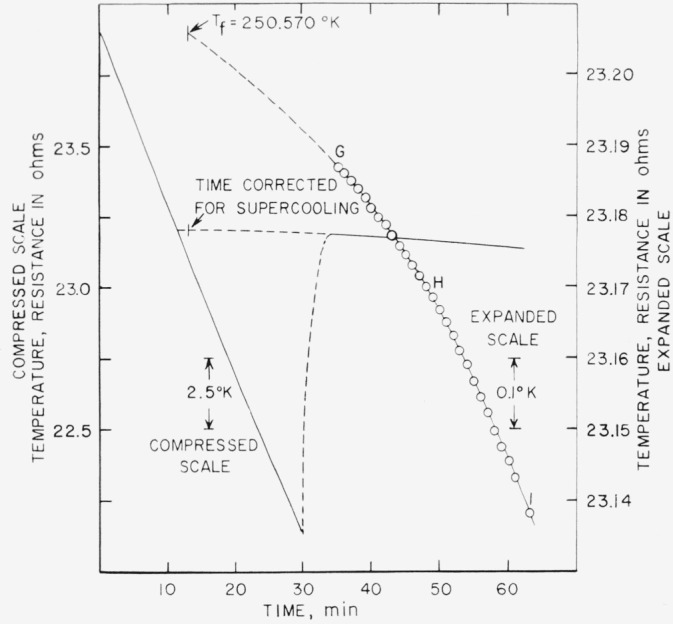
Freezing curve of methylphosphonyl chlorofluoride at saturation pressure.

**Figure 4:**
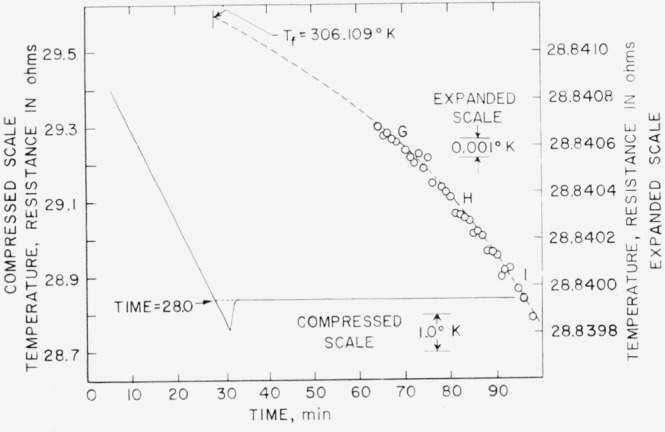
Freezing curve of methylphosphonyl dichloride at atmospheric pressure.

The results of the freezing-curve experiments are summarized in table 1. Figures 1, 2, and 3 show the freezing curves for difluoro, dichloro, and chlorofluoro, respectively. In the case of dichloro, measurements were also made at 1-atm pressure of dry air. For these measurements, air dried by cooling to liquid nitrogen temperature was admitted into the freezing-curve apparatus after the experiments were completed on the material at its saturation pressure. The results of these measurements are summarized at the bottom of table 1 and in figure 4. The purity and the freezing temperature are essentially unaffected by the presence of dry air. This indicates that dichloro is not decomposed by dry air and that the effects of the solution of air and of the pressure on the freezing temperature compensate each other.

The purity of chlorofluoro was not significantly improved by the purification treatment. The increase in purity was only 0.5 mole percent. This poor result is attributed to the presence of relatively higher melting (50 deg higher) dichloro as an impurity. Also the rate of disproportionation of chlorofluoro may be sufficiently high even at room temperature to yield difluoro and dichloro as impurities.

### 4.2. Calorimetric Method

The methylphosphonyl dihalide samples were received in glass break-seal ampoules after the completion of the determination of their purity and triple-point temperature by the freezing-curve method. The same precautions were taken as in the earlier handling processes in transferring the samples into the platinum-iridium vessel for the calorimetric measurements. The ampoule containing the sample was attached to a glass transfer manifold to which the calorimeter vessel was also attached by means of a platinum tube-soft glass seal. The platinum tube (⅛ in. O.D. × 0.007 in. wall and about ⅝ in. long) was attached to the calorimeter vessel by means of pure-gold solder. Prior to the transfer of the sample from the ampoule, the vessel and the connecting glass transfer manifold were heated to about 50 °C and evacuated to 10^−5^ torr or lower pressure for 48 hr in order to remove as much of the moisture adsorbed on the apparatus as possible. The glass transfer manifold was connected to the vacuum system via a glass stopcock lubricated with fluorocarbon grease which was previously baked in a vacuum oven at 150 °C. After the sample was transferred into the vessel by vacuum distillation, it was cooled to about 200 °K and pumped to remove any hydrogen halide that may have formed during storage from any small quantities of moisture adsorbed on the ampoule. The vessel was sealed at the glass tube as close as possible to the platinum-soft glass seal. The glass tube was previously thickened and constricted to facilitate the sealing under vacuum. At the end of transfer of each sample a small quantity of relatively less volatile oil-like material remained in the ampoule. No attempt was made to identify this residue.

During the process of sealing the calorimeter vessel after transferring the difluoro sample, a small crack appeared in the soft glass seal on the platinum tube. Although the crack was quickly fused, there is a possibility that a small amount of air entered and was sealed in the vessel. Because of this possibility, three sets of purity determinations were made—before, during, and after the heat-capacity measurements. The amount of air was, however, not sufficient to cause a “hump” in the heat capacity from the melting of nitrogen around 63 °K. (The sample vessel was approximately 50 percent full. One atmosphere of air in the vessel should cause a noticeable “hump” in the heat capacity.)

The procedures and principles involved in the calorimetric determination of purity and triple-point temperature have been described previously [3]. Briefly, the equilibrium melting temperatures are measured at various known liquid-to-solid ratios as determined from the successive measured increments of energy introduced, heat of fusion, and heat capacity. The observed equilibrium melting temperatures are plotted as the function of 1/*F*, the reciprocal of the fraction in the liquid state. A linear equation is fitted to the observations by the method of least squares and the product of the slope (*m*) of this equation and the cryoscopic constant (*A*) is taken to be the mole fraction impurity. The temperature intercept (*1*/*F*=0) is the triple point $\left(T_{t\;p}^{{}^{\circ}}\right)$ of the pure material.

**Figure 5:**
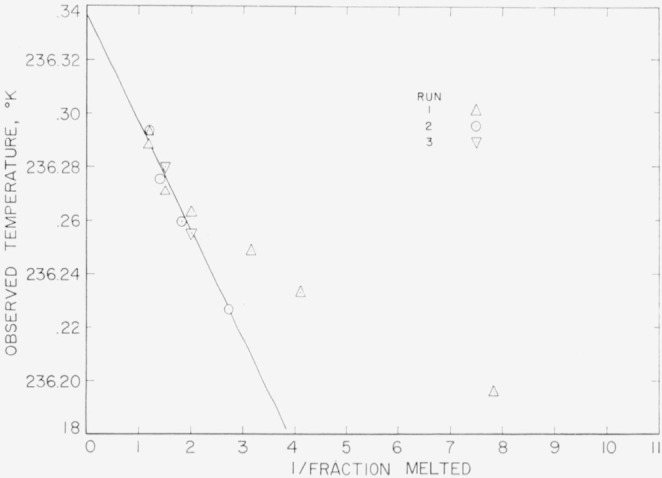
Equilibrium melting temperatures of methylphosphonyl difluoride at various reciprocals of liquid fraction.

**Figure 6:**
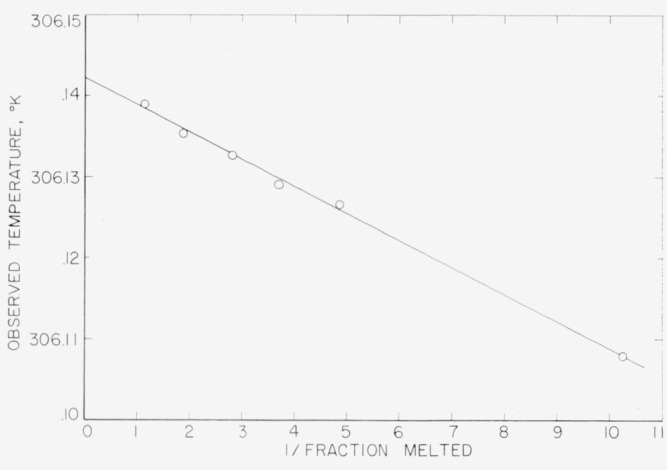
Equilibrium melting temperatures of methylphosphonyl dichloride at various reciprocals of liquid fraction.

**Figure 7:**
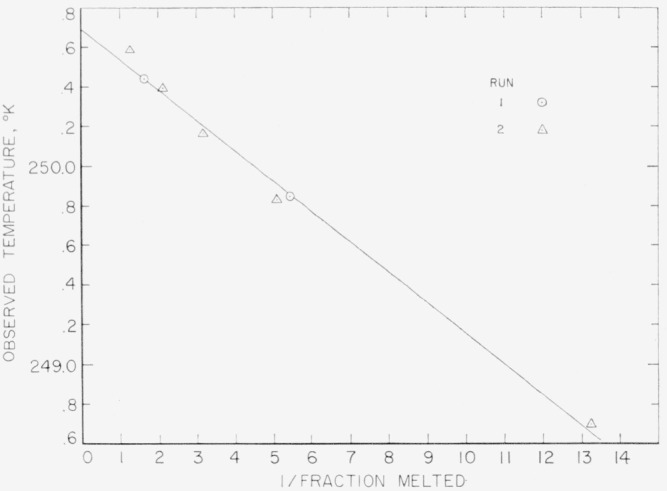
Equilibrium melting temperatures of methylphosphonyl chlorofluoride at various reciprocals of liquid fractions.

The results of the measurements of equilibrium melting temperatures are summarized in tables 2, 3, and 4 and in figures 5, 6, and 7 for difluoro, dichloro, and chlorofluoro, respectively. The temperatures in the column headed *T*_calc_ were obtained from the relation $T_{\text{calc}}=T_{t\;p}^{{}^{\circ}}-N_{2}/AF=T_{t\;p}^{{}^{\circ}}-m/F$, where *m* is the slope referred to in the above paragraph of the equation obtained by the method of least squares. After the mole fraction impurity (*N*_2_*=mA, where A* is the cryoscopic constant) and the triple-point temperature $T_{t\;p}^{{}^{\circ}}$ of the pure material were determined, *T*_calc_ was obtained at the various observed values of 1/*F.* The deviations between *T*_obs_ and *T*_calc_ indicate the combined effect of experimental error and non-ideal behavior of the system.

The estimates of uncertainties given for the triple-point temperatures and the impurity contents of the samples were obtained by examining the possible errors in the measurements of temperatures and of 1/*F* and by considering the possibility of equilibrium not having been attained under the conditions of the measurements.

The purity determinations on difluoro require further explanation. Since the sample was possibly contaminated by air and moisture, the purity of the sample was closely followed. The sample was in the calorimeter vessel a total of 16 days. On the 1st day, measurements of the heat capacity and heat of fusion were made and the sample recrystallized for the first purity determination on the following day. In the measurements of the 1st day the temperature of the sample was not raised above 270 °K. The purity determinations of run 2 were obtained on the 14th day following a series of heat-capacity and heat-of-fusion measurements in which the sample was not raised above room temperature. On the 15th day heat-capacity measurements were made up to about 340 °K. The purity determinations of run 3 were then made on the 16 th day.

The results of runs 2 and 3 show that the purity did not change significantly between the 14th and the 16th day although the temperature was raised in the meantime up to 340 °K. Except for the first three observations (1/*F*=7.84, 4.11, and 3.12), the results of run 1 are in fair agreement with those of the subsequent runs. The deviation of the above first three points (these were not used in the least squares analysis) may be the results of observations of nonequilibrium temperatures or the material and the impurities do not form an ideal solution. The deviation is considered due most likely to nonequilibrium conditions. On the basis of the above results and interpretation, the purity of the sample did not change during the 16 days of measurements. The higher purity obtained for the sample in the freezing-curve method indicates that some contamination did occur when the soft glass seal cracked.

### 4.3. Discussion of the Results

The results of the purity and triple-point temperatures obtained by the two methods are compared in table 5. A recent work at the NBS on the intercomparison of purity determination of benzene by freezing-curve and calorimetric methods showed that when the measurements are performed carefully using scrupulously cleaned equipment the two methods yield the same results [3, 15]. Because of the highly reactive nature of the substances investigated, the degree of dryness of the apparatus is expected to influence the purity results obtained. The hydrolysis products are observed as impurities. In the intercomparison studies with benzene an auxiliary pure sample was used to purge the apparatus before the sample to be investigated was introduced. Since relatively small amounts of purified samples were available, the above precautionary procedure was not followed with the methylphosphonyl dihalide samples.

The products of the hydrolysis reaction are widely different in volatility from the parent substance, therefore they are easily separated by distillation. The higher purity values obtained by the calorimetric method on the samples of dichloro and chlorofluoro indicate that the platinum-iridium calorimeter vessel was drier than the glass freezing-curve apparatus. The sample was purified in the process of transferring from the glass freezing-curve apparatus to the break-seal ampoule for storage. Impurities were produced by the moisture in the ampoule (as indicated by the less volatile residue referred to earlier), but they were removed again in the process of transfer to the calorimeter vessel.

The lower purity obtained by the calorimetric method on the difluoro sample shows that when the soft glass seal cracked some moisture entered and produced impurities. The purity was still sufficiently high to continue the heat-capacity measurements.

The chlorofluoro sample was thought originally to disproportionate to a high concentration of difluoro and dichloro. The calorimetric purity determination on chlorofluoro was done more than 1 year after the freezing-curve measurements, but the results show that the purity of the material had not changed significantly during this period while it was stored at room temperature. The results of run 2 in the calorimetric measurements, obtained after the sample was raised previously to 340 °K, show essentially the same purity as run 1 which was obtained before subjecting the sample to the higher temperatures. These results indicate that the disproportionation to a high concentration of difluoro and dichloro is a relatively slow process at room temperature and at 340 °K. On the other hand, if the equilibrium concentration is reached very rapidly, even at the triple-point temperature, the equilibrium dichloro and difluoro “impurities” in chlorofluoro will not be discernible in the cryoscopic purity determinations described in this paper.

The triple-point temperatures obtained on dichloro by the two methods are the same. The result obtained by the calorimetric method on difluoro of slightly lower purity is higher than that obtained by the freezing-curve method on a sample of higher purity. Conversely, the triple-point temperature obtained by the freezing-curve method on chlorofluoro of lower purity is higher than that obtained by the calorimetric method on a sample of higher purity. A recent systematic measurement at the NBS on a series of four benzene samples of decreasing purity indicates a possible trend toward an increasing triple-point temperature [15]. This suggests that the cryoscopic method for determining purity and triple-point temperature requires further study to investigate the factors that lead to these results.

## 5. Heats of Fusion

The heats of fusion of the samples were determined in the usual manner by measuring the amount of electrical energy needed to heat from a temperature below the triple-point to a temperature above it. Corrections were applied for the heat capacity of the container and sample below and above the triple point and for the premelting of the sample due to the presence of impurities. Because of the low purity of the difluoro and chlorofluoro samples, the electrical energy was introduced from a temperature considerably below the triple-point temperature in order to minimize the relatively uncertain premelting correction. The results of the measurements are summarized in tables 6, 7, and 8 for the difluoro, dichloro, and chlorofluoro samples, respectively. Although the precision of the measurements is shown to be fairly good, the uncertainties in the premelting corrections and in the heat capacities in the region below the triple-point temperature, in particular for the difluoro and chlorofluoro samples, impose large estimated uncertainties on the heats of fusion. With materials of low purity the absolute uncertainty is greater in the determination of the triple-point temperature and of the purity, which leads to a greater uncertainty in the determination of the premelting correction and in the heat capacity. The uncertainty of the solution behavior of the impurities contributes also to the overall uncertainty. The overall heat input is determined, however, with high accuracy limited only by the experimental equipment.

## 6. Heat Capacity

The heat capacity of the substances was measured from about 15 to 340 °K. Two sets of measurements, one on the vessel-plus-sample and the other on the empty vessel, were made on all three samples. The vessel-plus-sample measurements were completed first and the sample was removed by vaporization from the vessel through a small hole punched into the thin-wall platinum tube previously described. The vessel was thoroughly cleaned and the empty-vessel measurements were then made. For the subsequent sample a new platinum tube-soft glass seal was attached to the vessel by means of gold solder as previously described.

In order to minimize the curvature correction in the region where the heat capacity has a large curvature, the temperature increment of heating was in general smaller than 2 deg below 35 °K, 2 to 5 deg from 35 °K to about 90 °K, and as high as 8 to 10 deg at higher temperatures. Curvature corrections were applied wherever significant according to the relation given in a different notation by Osborne et al. [9]:

$$Z_{Tm}=Q/\Delta T-{\left(\partial^{2}Z/\partial T^{2}\right)}_{Tm}\left(\Delta T^{2}/24\right)\ldots$$ (1)

in which *Z_Tm_* is the corrected heat capacity at the mean temperature *T_m_* of the heating interval Δ*T; Q* is the electrical energy introduced; and (*∂^2^Z*/*∂T^2^*)*_Tm_* is the second derivative of the heat capacity with respect to temperature at *T_m_.* The values of (*∂^2^Z*/*∂T^2^*)*_Tm_* were obtained appropriately from a plot of (*∂^2^*/*∂T^2^*)(*Q*/Δ*T*) which is adequate for the purpose.

A smoothed table of heat-capacity values for the empty-vessel measurements was calculated at evenly spaced integral temperatures for the complete temperature range of the experiments. The observed heat capacity of the sample was obtained by first making curvature corrections to the observed mean heat capacity of the vessel-plus-sample experiments, then subtracting the heat capacity of the empty vessel obtained by interpolation in the smoothed table mentioned above.

The observed values of heat capacity given in tables 9, 10, and 11 for difluoro, dichloro, and chlorofluoro, respectively, were obtained by the above process and are expressed on a molal basis. The values listed have been corrected, in accordance with the method outlined by Hoge [5], for vaporization effects down to temperatures where the corrections become insignificant. Vapor pressure and density data for the corrections were taken from those reported by Zeffert, Coulter, and Tannenbaum [16]. The maximum vaporization corrections for difluoro, dichloro, and chlorofluoro were 0.45, 0.43, and 0.08 percent, respectively, at the highest temperature of the observations.

**Figure 8:**
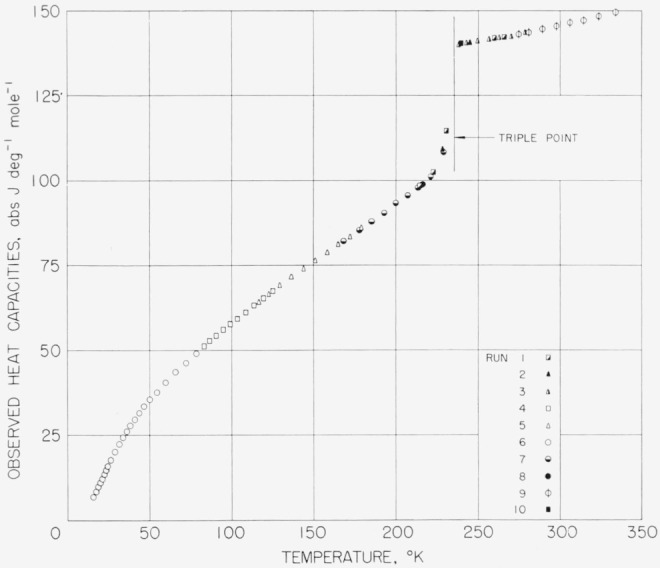
Observed heat capacity of methylphosphonyl difluoride.

**Figure 9:**
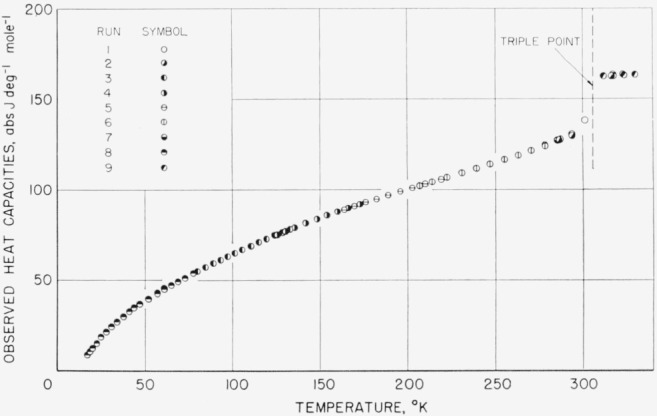
Observed heat capacity of methylphosphonyl dichloride.

**Figure 10:**
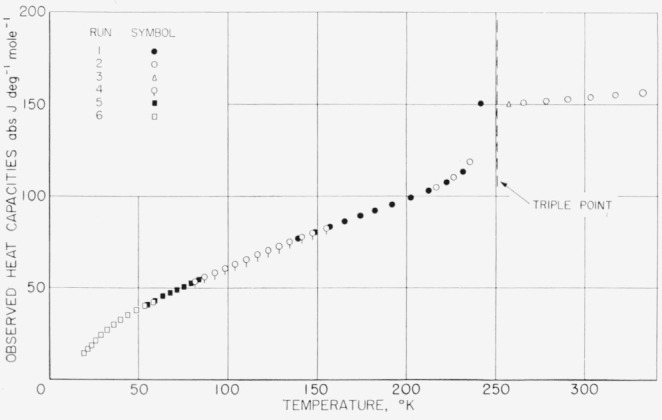
Observed heat capacity of methylphosphonyl chlorofluoride.

The values listed, however, have not been corrected for premelting effects in the region below the triple-point temperature. Corrections for curvature and vaporization have been applied to the given values of observed heat capacity, because these corrections are dependent upon the experimental conditions. The premelting correction is, however, dependent only upon the nature of the substance including the impurity. Figures 8, 9, and 10 show plots of the observed values of heat capacity of the methylphosphonyl dihalides.

The observed values of the heat capacity were not corrected for the contribution of the impurities. The heat capacity of the impurities was assumed to be the same as the sample.

Smoothed values of heat capacity were calculated at evenly spaced integral temperatures for each of the substances from the analysis of the observed values of heat capacity given in tables 9, 10, and 11. The values in the region just below the triple-point temperature were obtained by applying premelting corrections and by judiciously extrapolating the values at the lower temperatures to the triple-point temperature. Those values of heat capacity very close to the triple-point temperature are believed to be uncertain by as much as 2 to 3 percent for difluoro and chlorofluoro. The values for the purer dichloro are considered to be more accurate. Measurements of the experimental heat input are believed to be accurate to about 0.01 percent. The analysis of the experimental data has been performed so that the *fCdT* plus *L_f_* would be consistent with the above accuracy. Thus, although the distribution of the heat input to the heat capacity and to the heat of fusion may have a high uncertainty, the sum of the two heat inputs is expected to be accurate to 0.01 percent.

The final smoothed values of molal heat capacity are given in tables 12, 13, and 14 for difluoro, dichloro, and chlorofluoro, respectively.

## 7. Derived Thermal Properties

Values of relative enthalpy, enthalpy function, entropy, Gibbs free energy, and Gibbs free energy function were calculated from the smoothed values of heat capacity by tabular integration, using Lagrangian four-point integration coefficients. The thermodynamic relations used are as follows:

$$\left(H_{\text{Sat}}-H_{0}^{c}\right)=\int_{0}^{T}C_{\text{Sat}}dT+\int_{0}^{T}V_{\text{Sat}}\left(dp/dT\right)dT,$$ (2)

$$\left(S_{\text{sat}}-S_{0}^{c}\right)=\int_{0}^{T}\left(C_{\text{sat}}/T\right)dT,$$ (3)

$$-\left(G_{\text{sat}}-H_{0}^{c}\right)=T\left(S_{\text{sat}}-S_{0}^{c}\right)-\left(H_{\text{sat}}-H_{0}^{c}\right)+TS_{0}^{c}$$ (4)

The relation:

$$-\left(G_{\text{sat}}-H_{0}^{c}\right)=\int_{0}^{T}\left(S_{\text{sat}}-S_{0}^{c}\right)dT-\int_{0}^{T}V_{\text{sat}}\left(dp/dT\right)dT+TS_{0}^{c}$$ (5)

was also evaluated to check the calculations in eq (4). In the evaluation of the above expressions, the residual entropy $\left(S_{0}^{c}\right)$ was taken to be zero and adjustments were made for the phase transitions (heat of fusion) wherever necessary. The enthalpy function and the Gibbs free energy function were obtained by dividing values of enthalpy and Gibbs free energy, respectively, by the appropriate temperatures. The values of heat capacity below the experimental range of each substance were obtained by extrapolation using a Debye heat-capacity function fitted to the experimental values obtained at the lower temperatures. The evaluation of the thermal properties was done on the IBM 7090 computer at 1-deg intervals up to 75 °K and at 5-deg intervals at the higher temperatures. The calculated values were rounded to three decimals or rounded to five significant figures for the larger values. Smoothed values of heat capacity and the derived thermal functions are given at 5-deg intervals in tables 12, 13, and 14.

## 8. Discussion of Results

**Figure 11:**
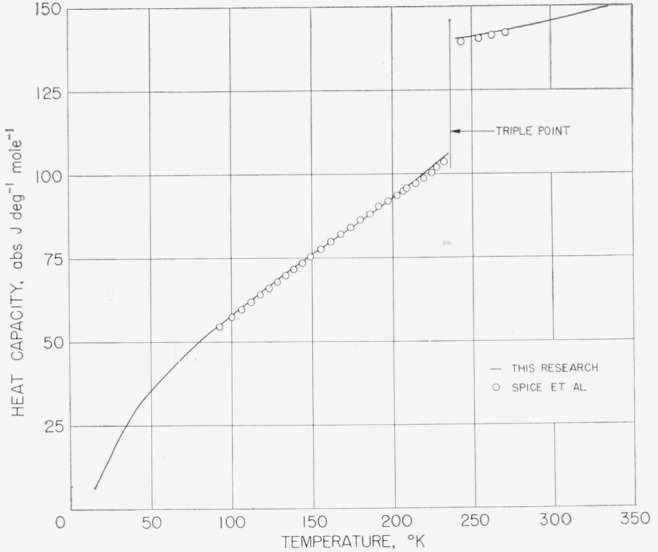
Comparison of the observed heat capacity of methylphosphonyl difluoride with those reported by Spice et al. [11].

**Figure 12:**
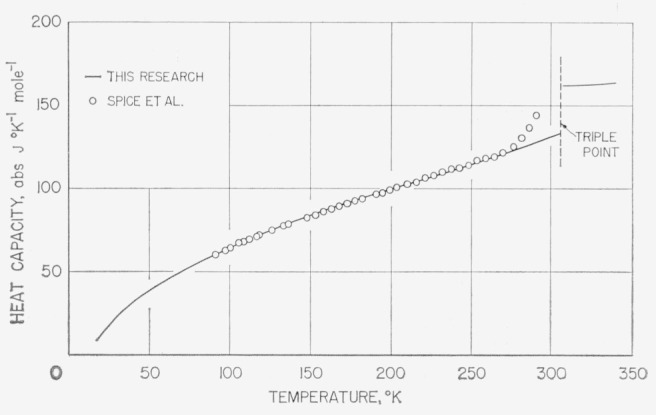
Comparison of the observed heat capacity of methylphosphonyl dichloride with those reported by Spice et al. [11].

At about the time of the conclusion of the measurements, the authors noticed in unpublished British literature heat-capacity measurements on difluoro and dichloro in the range of about 90 to 290 °K by Spice et al. [11]. In figures 11 and 12 are compared the results on difluoro and dichloro, respectively.^5^ The difluoro samples were of comparable purity while the dichloro sample investigated by Spice was considerably less pure. The lower purity is quite evident from the relatively large premelting effects shown, the values not having been adjusted for premelting. For difluoro, Spice gave values of heat capacity adjusted for premelting. These adjusted values are shown to be in close agreement with the final smoothed values selected in the present investigation (solid curve).

Spice calculated the entropy of gaseous difluoro and dichloro at 298.15 °K from his data by extrapolating below 90 °K and by estimating the heat of vaporization at 298.15 °K using vapor-pressure data and heat-of-vaporization measurements at higher temperatures given in unpublished literature. These results are compared in table 15 with those obtained in the present work. In the present work the heat of vaporization was estimated using the Clausius-Clapeyron relation and vapor-pressure equations for difluoro, dichloro, and chlorofluoro given by Zeffert et al. [16]. For the dichloro compound, the vapor-pressure equation for the liquid was extrapolated down to 298.15 °K. The heat of fusion at 298.15 °K was calculated assuming constant difference in the heat capacity of the liquid and solid between the triple-point temperature (306.14 °K) and 298.15 °K, the experimentally observed difference at the triplepoint temperature being used. The calculation of the entropy of chlorofluoro is also given in table 15. The tables show that the experimental work between 90 and 298.15 °K are in close agreement. The extrapolation by Spice et al., [11] below 90 °K gives entropy values about 6 J/deg-mole lower than the present experimentally obtained values with extrapolation only below 15 °K which contributes only about 2 J/deg mole total.

The difference in the entropy of vaporization for difluoro is consistent with the possible error of 1 to 2 percent often present in calculating the heat of vaporization using Clausius-Clapeyron equation and vapor-pressure data. The large difference in the entropy of sublimation for dichloro arises largely from the low value obtained by Spice for the heat of fusion. Spice considered the measurements in the region of the melting point of dichloro beyond the upper temperature limit of his calorimeter for accurate measurements. Using the experimentally observed heat of fusion of the present work with Spice’s value for the heat of vaporization, the entropy of sublimation would become 221.3 J/deg-mole, which is closer to that value obtained in the present work. This comparison of entropy values shows that the entropy of vaporization could be estimated within 1 to 2 percent from vapor-pressure data. The extrapolation below 90 °K could, however, introduce a much larger error.

## Figures and Tables

**Table 1:** Summary of purification conditions and results of purity and triple-point temperature determinations of methylphosphonyl difluoride, dichloride, and chlorofluoride by the freezing-curve method

Freezing point at saturation pressure	Freezing point if no impurity present	Purity	Cryoscopic constant used	Temperature and temperature bath used during purification	
				Crystallization temperature	Melting temperature
Methylphosphonyl difluoride					
*°C.* a−36.86±0.01	*°C.* a−36.84±0.01	*Mole* % a99.95±0.01	0.0256	*°C* −78 Dry ice slush	*°C* −30 CC*l*_4_ – CHC*l*_3_ Mixture cooled with dry ice.
Methylphosphonyl dichloride (saturation pressure)					
32.96±0.01	32.99±0.01	99.92±0.01	0.0232	+25 Constant temperature room.	+38 Heating tape
Methylphosphonyl dichloride (1 atm pressure)					
32.95±0.01	32.99±0.01	99.91 ±0.01	0.0232	+25 Constant temperature room.	+38 Heating tape.
Methylphosphonyl chlorofluoride					
−22.5±0.1	−22.1±0.1	99.0±0.1	0.0228	−78 Dry ice slush.	−15 CC*l*_4_–CHC*l*_3_ Mixture cooled with dry ice.

a The figures preceded by ± indicate estimated uncertainty.

**Table 2:** Equilibrium melting temperatures of methylphosphonyl difluoride °K=°C + 273.15° $N_{2}=0.0256\left(T_{\text{t p}}^{{}^{\circ}}-T_{\text{obs}}\right)$

Run No.	1/*F*^a^	*T*_obs_	*T*_calc_

1	7.84	b 236.1962	235.0193
	4.11	.2329	.1711
	3.12	.2483	.2114
	2.02	.2624	.2562
	1.49	.2728	.2778
	1.18	.2891	.2904
2	2.72	.2269	.2277
	1.88	.2601	.2619
	1.43	.2762	.2802
	1.16	.2936	.2912
3	1.99	.2564	.2574
	1.48	.2798	.2782
	1.18	.2936	.2904
	1.00	……	.2977
	0.00	……	.3384
Triple-point temperature, 236.34±0.02 °K.^c^ Slope, −0.0407 °K. Impurity, 0.11±0.10 mole percent.^c^			

a *F* is the fraction of sample melted.

b The temperatures given are believed to be accurate to ±0.01 °K. Wherever temperatures are expressed to the fourth decimal place, the last two figures are significant only insofar as small temperature differences are concerned.

c The uncertainty was estimated by examining the imprecision of the measurements and the various known sources of systematic error. The substance is assumed to obey Raoult’s law of solution and to form no solid solution with the impurities present.

**Table 3:** Equilibrium melting temperatures of methylphosphonyl dichloride °K = °C + 273.15° $N_{2}=0.0232\left(T_{t\;p}^{{}^{\circ}}-T_{\text{obs}}\right)$

1/*F*^a^	*T*_obs_	*T*_calc_

10.25	b 306.1080	306.1080
4.85	.1267	.1260
3.68	.1292	.1299
2.79	.1327	.1328
1.88	.1356	.1358
1.10	.1389	.1384
1.00	……	.1388
0.00	……	.1421
Triple-point temperature, 306.14±0.02 °K.^c^ Slope, −0.00322 °K. Impurity, 0.008±0.005 mole percent.^c^		

a See footnote a, table 2.

b See footnote b, table 2.

c See footnote c, table 2.

**Table 4:** Equilibrium melting temperatures of methylphosphonyl chlorofluoride °K = °C + 273.15° $N_{2}=0.0228\left(T_{t\;p}^{{}^{\circ}}-T_{\text{obs}}\right)$

Run No.	1/F^a^	*T*_obs_	*T*_calc_

1	5.45	b 249.8502	249.8570
	1.63	250.4456	250.4445
2	13.25	248.6941	248.6574
	5.10	249.8255	249.9108
	3.13	250.1608	250.2138
	2.10	250.3980	250.3722
	1.25	250.5854	250.5030
	1.00	……	250.5414
	0.00	……	250.6952
Triple-point temperature, 250.70±0.20 °K.^c^ Slope, −0.1538 °K. Impurity, 0.35±0.10 mole percent.^c^			

a See footnote a, table 2.

b See footnote b, table 2.

c See footnote c, table 2.

**Table 5:** Comparison of the purities and triple-point temperatures of methylphosphonyl difluoride, methylphosphonyl dichloride, and methylphosphonyl chlorofluoride samples obtained by the freezing-curve and calorimetric methods

Compound	Purity		Triple-point temperature	

	Freezing-curve	Calorimetric	Freezing-curve	Calorimetric

	*Mole* %	*Mole* %	*°K*	*°K*
Difluoro	a 99.95±0.01	a 99.89 ±0.10	a 236.31 ±0.01	a 236.34±0.02
Dichloro	99.92±0.01	99.992±0.005	306.14±0.01	306.14±0.02
Dichloro^b^	99.91 ±0.01	……	306.14±0.01	……
Chlorofluoro	99.0 ±0.1	99.65 ±0.10	251.1 ±0.1	250.70±0.20

a See footnote c, table 2.

b Saturated with dry air and at 1 atm pressure.

**Table 6:** Molal heat of fusion of methylphosphonyl difluoride Gram molecular weight=100.0051 g, mass of sample=37.8505 g Triple-point temperature=236.34 °K, °K=°C + 273.15°

Temperature interval	Heat input^a^	Heat capacity and premelting corrections^a^	Δ*H*^b^	*L_f_*

*°K*	*J*	*J*	*J*	*J*/*mole*
218.8406 to 256.5282	7244.6	−2748.9	4495.7	11878
222.4604 to 241.6295	5820.1	−1324.3	4495.8	11878
215.6684 to 237.3642	5912.3	−1418.7	4493.6	11872
217.3546 to 239.6000	5987.5	−1489.6	4498.0	11884
174.5253 to 237.7456	8396.4	−3900.3	4496.1	11879

Mean				11878
Standard deviation of the mean^c^				±2
Estimated uncertainty of the mean^d^				±12

a Includes enthalpy change associated with container.

b The heat of melting for 37.8505 g of sample.

c Standard deviation of the mean as used here is defined as [Σd2/n(n−1)]12 where *d* is the difference of a single observation from the mean and *n* is the number of observations.

d See footnote c, table 2.

**Table 7:** Molal heat of fusion of methylphosphonyl dichloride Gram molecular weight = 132.914 g, mass of sample=34.6223 g Triple-point temperature=306.14 °K, °K=°C + 273.15°

Temperature interval	Heat input^a^	Heat capacity and premelting corrections^a^	Δ*H*^b^	*L_f_*

*°K*	*J*	*J*	*J*	*J*/*mole*
297.4007 to 314.4917	5838.7	−1,128.9	4709.8	18,081
298.6786 to 312.7249	5632.0	−925.2	4706.7	18,070
298.2602 to 308.8547	5388.2	−679.4	4708.8	18,077

Mean				18 076
Standard deviation of the mean^c^				±3
Estimated uncertainty of the mean^d^				±15

a See footnote a, table 6.

b The heat of melting for 34.6223 g of sample.

c See footnote c, table 6.

d See footnote c, table 2.

**Table 8:** Molal heat of fusion of methylphosphonyl chlorofluoride Gram molecular weight=116.460 g, mass of sample=27.0127 g Triple-point temperature=250.70 °K, °K=°C + 273.15°

Temperature interval	Heat input a	Heat capacity and premelting corrections^a^	Δ*H*^b^	*L_f_*

*°K*	*J*	*J*	*J*	*J*/*mole*
207.5262 to 258.4266	5412.8	−2665.3	2747.5	11845
211.7855 to 258.9488	5241.9	−2488.1	2753.8	11872
212.5767 to 254.8591	4952.3	−2197.2	2755.1	11878
161.5768 to 258.0041	7500.9	−4759.9	2741.0	11817

Mean				11853
Standard deviation of the mean^c^				±14
Estimated uncertainty of the mean^d^				±30

a See footnote a, table 6.

b The heat of melting for 27.0127 g of sample.

c See footnote c, table 6.

d See footnote c, table 2.

**Table 9:** Observed molal heat capacity of methylphosphonyl difluoride Gram molecular weight=100.0051 g, °K=°C + 273.15°

Run No.	*T_m_*^a^	Δ*T*	*C*_sat_^b^

	*°K*	*°K*	*J*/*deg-mole*
1	c 214.7574	c 8.1664	98.390
	222.8022	7.9233	102.18
	230.4280	7.3282	114.44
	259.6332	6.2101	141.71
	265.8382	6.1997	142.19
2	228.0814	11.2420	109.16
	244.8004	6.3418	140.61
3	238.4352	2.1420	140.10
	242.8167	6.6210	140.51
	249.4139	6.5734	140.95
	255.9706	6.5399	141.43
	262.4992	6.5174	141.87
	270.0368	8.5577	142.56
	278.5608	8.4903	143.29
4	83.1686	3.5007	51.024
	86.6180	3.3981	52.545
	90.5122	4.3902	54.068
	94.8333	4.2520	55.742
	99.0247	4.1308	57.351
	103.5945	5.0088	59.110
	108.5268	4.8557	60.986
	113.7826	5.6560	62.982
	119.3510	5.4808	65.061
	125.1973	6.2091	67.209
5	116.7778	4.6351	64.098
	122.7352	6.2795	66.312
	129.6306	6.2107	68.852
	136.5044	7.5367	71.365
	143.9268	7.3083	73.808
	151.1290	7.0960	76.288
	158.1372	6.9203	78.676
	164.9730	6.7514	80.957
	172.2738	7.8501	83.299
	179.0744	5.7512	85.760
6	15.6738	1.4849	6.8428
	17.2192	1.6059	8.3430
	18.6956	1.3469	9.8400
	19.9582	1.1784	11.146
	21.1345	1.1742	12.368
	22.3080	1.1727	13.431
	23.4228	1.0570	14.680
	24.6048	1.3070	15.843
	26.4544	2.3923	17.693
	29.0166	2.7320	20.083
	31.5850	2.4048	22.336
	33.8582	2.1416	24.344
	35.9750	2.0921	26.087
	38.2220	2.4017	27.794
	40.8473	2.8490	29.638
	43.6756	2.8076	31.532
	46.6350	3.1112	33.409
	49.9479	3.5146	35.374
	54.2472	5.0841	37.609
	59.5584	5.5393	40.361
	65.7194	6.7825	43.373
	72.2326	6.2441	46.194
	78.4064	6.1035	48.888
7	168.1723	11.8746	81.972
	177.9294	7.6395	85.196
	185.4762	7.4541	87.736
	192.8409	7.2658	90.279
	200.0195	7.0914	93.147
	207.0449	6.9594	95.398
	213.9396	6.8300	97.925
	221.1040	7.4989	101.12
	228.8298	7.9527	108.14
8	216.0872	8.0162	98.797
9	274.5950	2.8771	142.95
	280.3669	8.6666	143.49
	289.0014	8.5971	144.31
	297.5207	8.4416	145.12
	305.9286	8.3743	145.98
	314.2738	8.3159	146.78
	323.5867	10.3100	147.81
	333.8511	10.2188	148.89
10	238.9768	2.4624	140.22

a *T_m_* is the mean temperature of the heating interval Δ*T.*

b *C*_sat_ is the heat capacity of the condensed phase at its saturation pressure corresponding to *T_m_*

c See footnote b, table 2.

**Table 10:** Observed molal heat capacity of methylphosphonyl dichloride Gram molecular weight=132.914 *g*, °K=°C + 273.15°

Run No.	*T_m_*^a^	Δ*T*	*C*_sat_^b^

	*°K*	*°K*	*J*/*deg-mole*
1	c 278.6232	c 10.5053	124.10
	287.2900	6.8285	127.51
	294.0525	6.6964	130.25
	301.4278	8.0542	138.12
	317.3858	8.7881	162.96
	323.1628	5.7660	163.36
2	286.5885	24.1802	127.09
	315.6274	5.8049	162.56
3	79.9972	4.7091	54.737
	84.5806	4.4577	57.168
	88.9450	4.2709	59.258
	93.1406	4.1204	61.185
	97.2455	4.0894	62.975
	101.2782	3.9759	64.762
	105.6774	4.8181	66.683
	110.4212	4.6694	68.725
	115.0274	4.5429	70.701
	119.5879	4.5782	72.589
	124.1132	4.4723	74.458
	128.5400	4.3813	76.187
	132.8765	4.2918	77.880
4	125.7240	4.5228	75.026
	130.1903	4.4098	76.824
	135.6236	6.4567	78.898
	142.0402	6.2902	81.298
	148.2580	6.1453	83.529
	154.3394	6.0175	85.664
	160.2976	5.8989	87.638
	166.4835	6.4730	89.662
	172.8980	6.3561	91.758
5	163.8636	5.4478	88.782
	169.7947	6.4144	90.764
	176.1486	6.2935	92.780
	182.3872	6.1810	94.641
	188.9614	6.9673	96.722
	195.8652	6.8405	98.782
	202.6528	6.7347	100.66
	210.4300	8.8196	103.06
	219.1628	8.6461	105.58
6	207.0185	5.4714	102.05
	214.0554	8.6019	104.12
	222.5762	8.4397	106.62
	230.9417	8.2918	109.04
	239.1618	8.1391	111.48
	247.2356	8.0084	113.90
	255.1850	7.8905	116.28
	263.0122	7.7637	118.71
	270.7074	7.6269	121.28
	278.2708	7.4997	123.89
	285.6995	7.3578	126.87
7	56.9483	4.1918	42.719
	60.9897	3.8910	44.975
	64.9204	3.9704	47.191
	68.9392	4.0673	49.153
	72.9073	3.8688	51.134
	77.5812	5.4791	53.482
8	17.0568	1.5970	8.647
	18.5078	1.3051	10.457
	19.9837	1.6466	12.239
	22.3783	3.1426	15.123
	25.1980	2.4969	18.369
	27.9984	3.1039	21.388
	30.8674	2.6341	24.280
	33.9492	3.5294	27.099
	37.3522	3.2766	30.094
	40.7683	3.5556	32.621
	43.6298	2.1675	34.593
	47.1684	4.9095	36.915
	51.8185	4.3908	39.804
	56.4084	4.7891	42.420
	61.0038	4.4016	44.994
9	294.0789	8.3626	130.11
	311.8420	5.9693	162.48
	317.8000	5.9465	162.67
	323.7360	5.9257	162.86
	329.6517	5.9056	163.16

a See footnote a, table 9.

b See footnote b, table 9.

c See footnote b, table 2.

**Table 11:** Observed molal heat capacity of methylphosphonyl chlorofluoride Gram molecular weight = 116.460 g, °K=°C +273.15°

Run No.	*T_m_*^a^	Δ*T*	*C*_sat_^b^

	*°K*	*°K*	*J*/*deg-mole*
1	c 139.7868	c 8.9624	76.997
	148.5676	8.5993	80.237
	157.0592	8.3839	83.235
	165.6160	8.7296	86.235
	174.2316	8.5016	89.171
	182.6321	8.2994	92.095
	192.0382	10.5127	95.382
	202.4104	10.2317	99.151
	212.5044	9.9564	103.01
	222.3294	9.6935	107.26
	231.8650	9.3778	113.12
	241.5778	10.0477	140.39
	248.2259	3.2486	670.13
2	216.5858	9.6007	104.74
	226.1950	9.6177	110.08
	235.6094	9.2109	118.76
	265.3440	12.7903	150.87
	278.0760	12.6739	151.64
	290.6889	12.5518	152.61
	303.1802	12.4307	153.51
	317.0766	15.3632	154.69
	332.3526	15.1887	155.87
3	249.2598	1.1314	1381.3
	250.4917	0.1874	2049.3
	252.7222	4.2737	700.28
	257.0449	4.3716	150.21
4	81.4146	5.6221	53.425
	86.8612	5.2713	55.950
	92.5048	6.0158	58.282
	98.3896	5.7537	60.701
	104.0347	5.5366	63.036
	110.0878	6.5697	65.484
	116.5386	6.3319	68.109
	122.7670	6.1249	70.543
	128.8026	5.9463	72.858
	134.6714	5.7911	75.107
	141.5162	7.8987	77.751
	147.4880	4.0449	79.918
	155.5436	12.0663	82.644
	252.4673	7.3741	1811.8
5	54.7214	3.7825	40.901
	58.8980	4.5707	43.042
	63.3030	4.2393	45.296
	67.4194	3.9935	47.182
	71.3158	3.7994	48.822
	75.1879	3.9448	50.569
	79.0498	3.7795	52.339
	83.2006	4.5220	54.278
6	19.2369	1.5626	14.260
	21.0195	2.0026	16.249
	23.1519	2.2622	18.550
	25.7266	2.8871	21.181
	28.7823	3.2244	24.070
	32.1024	3.4159	26.968
	35.5791	3.5374	29.713
	39.3997	4.1038	32.362
	43.6982	4.4933	34.984
	48.2508	4.6117	37.588
	53.2988	5.4843	40.249
	58.5218	4.9619	42.866

a See footnote a, table 9.

b See footnote b, table 9.

c See footnote b, table 2.

**Table 12:** Molal thermal functions for methylphosphonyl difluoride (*CH*_3_*POF*_2_) Gram molecular weight=100.0051 g, *T* deg K*=t* deg C + 273.15

*T*	*C*_sat_	$\left(H_{T}-H_{0}^{c}\right)$	$\frac{\left(H_{T}-H_{0}^{c}\right)}{T}$	*S_T_*	$-\left(G_{T}-H_{0}^{c}\right)$	$\frac{-\left(G_{T}-H_{0}^{c}\right)}{T}$
Solid						
*°K*	*J*/*deg*	*J*	*J*/*deg*	*J*/*deo*	*J*	*J*/*deg*
0.00	0.000	0.000	0.000	0.000	0.000	0.000
5.00	.264	.330	.066	.088	.110	.022
10.00	2.072	5.237	.524	.699	1.755	.175
15.00	6.124	24.946	1.663	2.242	8.684	.579
20.00	11.207	68.151	3.407	4.693	25.705	1.285
25.00	16.263	136.94	5.478	7.742	56.606	2.264
30.00	20.970	230.24	7.675	11.131	103.69	3.456
35.00	25.292	346.07	9.888	14.693	168.20	4.806
40.00	29.047	482.22	12.055	18.324	250.74	6.268
45.00	32.384	635.91	14.131	21.941	351.41	7.809
50.00	35.400	805.52	16.110	25.512	470.07	9.401
55.00	38.002	989.13	17.984	29.010	606.41	11.026
60.00	40.567	1185.6	19.759	32.427	760.03	12.667
65.00	42.992	1394.5	21.454	35.771	930.55	14.316
70.00	45.309	1615.3	23.076	39.042	1117.6	15.966
75.00	47.555	1847.5	24.633	42.245	1320.9	17.611
80.00	49.727	2090.7	26.134	45.383	1539.9	19.249
85.00	51.830	2344.6	27.584	48.462	1774.6	20.877
90.00	53.861	2608.9	28.988	51.482	2024.5	22.494
95.00	55.815	2883.1	30.349	54.447	2289.3	24.098
100.00	57.742	3167.0	31.670	57.359	2568.8	25.688
105.00	59.661	3460.5	32.957	60.222	2862.8	27.265
110.00	61.562	3763.6	34.215	63.042	3171.0	28.827
115.00	63.443	4076.1	35.445	65.820	3493.2	30.375
120.00	65.305	4398.0	36.650	68.559	3829.1	31.909
125.00	67.146	4729.1	37.833	71.263	4178.7	33.430
130.00	68.955	5069.4	38.995	73.932	4541.7	34.936
135.00	70.727	5418.6	40.138	76.567	4918.0	36.429
140.00	72.472	5776.6	41.262	79.171	5307.3	37.909
145.00	74.193	6143.3	42.368	81.744	5709.6	39.377
150.00	75.897	6518.5	43.457	84.288	6124.7	40.831
155.00	77.580	6902.2	44.531	86.804	6552.5	42.274
160.00	79.248	7294.3	45.589	89.294	6992.7	43.704
165.00	80.907	7694.7	46.634	91.758	7445.4	45.123
170.00	82.565	8103.4	47.667	94.198	7910.3	46.531
175.00	84.220	8520.3	48.688	96.615	8387.3	47.927
180.00	85.891	8945.6	49.698	99.011	8876.4	49.313
185.00	87.582	9379.3	50.699	101.39	9377.4	50.688
190.00	89.298	9821.5	51.692	103.75	9890.2	52.054
195.00	91.058	10272	52.679	106.09	10415	53.409
200.00	92.851	10732	53.660	108.42	10951	54.755
205.00	94.657	11201	54.638	110.73	11499	56.092
210.00	96.483	11679	55.613	113.03	12058	57.421
215.00	98.335	12166	56.585	115.33	12629	58.741
220.00	100.21	12662	57.555	117.61	13212	60.053
225.00	102.13	13168	58.524	119.88	13805	61.357
230.00	104.06	13683	59.493	122.15	14410	62.654
235.00	105.99	14208	60.462	124.40	15027	63.944
236.34	106.50	14351	60.721	125.01	15194	64.288
Liquid						
236.34	140.08	26230	110.99	175.27	15194	64.288
240.00	140.31	26744	111.43	177.43	15839	65.997
245.00	140.64	27446	112.02	180.32	16734	68.301
250.00	140.99	28150	112.60	183.17	17642	70.570
255.00	141.35	28856	113.16	185.96	18565	72.805
260.00	141.74	29564	113.71	188.71	19502	75.008
265.00	142.13	30273	114.24	191.42	20452	77.179
270.00	142.54	30985	114.76	194.08	21416	79.319
273.15	142.81	31434	115.08	195.73	22030	80.652
275.00	142.97	31699	115.27	196.70	22393	81.429
280.00	143.41	32415	115.77	199.28	23383	83.511
285.00	143.87	33133	116.26	201.82	24386	85.564
290.00	144.34	33854	116.74	204.33	25401	87.590
295.00	144.83	34577	117.21	206.80	26429	89.590
298.15	145.14	35033	117.50	208.34	27083	90.836
300.00	145.33	35302	117.67	209.24	27469	91.564
305.00	145.84	36030	118.13	211.64	28521	93.513
310.00	146.36	36761	118.58	214.02	29585	95.437
315.00	146.89	37494	119.03	216.36	30661	97.338
320.00	147.43	38230	119.47	218.68	31748	99.216
325.00	147.98	38969	119.90	220.97	32847	101.07
330.00	148.54	39710	120.33	223.23	33957	102.91
335.00	149.12	40455	120.75	225.47	35079	104.72

$H_{0}^{c}$ is the enthalpy of the crystal at the saturation pressure at 0 °K.

**Table 13:** Molal thermal functions for methylphosphonyl dichloride (CH_3_POCl_2_) Gram molecular weight = 132.914 g, *T* deg K *= t* deg C + 273.15

*T*	*C*_sat_	$\left(H_{T}-H_{0}^{c}\right)$	$\frac{\left(H_{T}-H_{0}^{c}\right)}{T}$	*S_T_*	$-\left(G_{T}-H_{0}^{c}\right)$	$\frac{-\left(G_{T}-H_{0}^{c}\right)}{T}$
Solid						
*°K*	*J*/*deg*		*J*/*deg*	*J*/*deg*	*J*	*J*/*deg*
0.00	0.000	0.000	0.000	0.000	0.000	0.000
5.00	.262	.328	.065	.088	.110	.022
10.00	2.071	5.208	.521	.695	1.743	.174
15.00	6.384	25.353	1.690	2.270	8.696	.580
20.00	12.266	71.691	3.584	4.895	26.217	1.311
25.00	18.133	147.90	5.916	8.272	58.904	2.356
30.00	23.416	252.04	8.401	12.055	109.61	3.654
35.00	28.045	380.96	10.884	16.020	179.75	5.136
40.00	32.054	531.45	13.286	20.033	269.89	6.747
45.00	35.532	700.62	15.569	24.014	380.03	8.445
50.00	38.663	886.20	17.724	27.922	509.91	10.198
55.00	41.604	1086.9	19.762	31.746	659.11	11.984
60.00	44.400	1302.0	21.700	35.487	827.23	13.787
65.00	47.098	1530.8	23.550	39.148	1013.8	15.598
70.00	49.711	1772.8	25.326	42.734	1218.6	17.408
75.00	52.287	2027.8	27.038	46.252	1441.1	19.214
80.00	54.831	2295.6	28.695	49.708	1681.0	21.012
85.00	57.325	2576.0	30.306	53.107	1938.1	22.801
90.00	59.727	2868.7	31.875	56.452	2212.0	24.577
95.00	61.994	3173.1	33.401	59.743	2502.5	26.342
100.00	64.201	3488.6	34.886	62.979	2809.3	28.093
105.00	66.407	3815.1	36.334	66.165	3132.2	29.830
110.00	68.579	4152.6	37.751	69.304	3470.9	31.553
115.00	70.701	4500.8	39.137	72.400	3825.2	33.262
120.00	72.783	4859.5	40.496	75.453	4194.8	34.957
125.00	74.795	5228.5	41.828	78.465	4579.6	36.637
130.00	76.764	5607.4	43.134	81.437	4979.4	38.303
135.00	78.684	5996.0	44.415	84.370	5393.9	39.955
140.00	80.558	6394.2	45.673	87.266	5823.0	41.593
145.00	82.399	6801.6	46.907	90.125	6266.5	43.217
150.00	84.182	7218.1	48.120	92.948	6724.2	44.828
155.00	85.905	7643.3	49.312	95.737	7195.9	46.425
160.00	87.593	8077.1	50.482	98.491	7681.5	48.009
165.00	89.240	8519.1	51.631	101.21	8180.8	49.581
170.00	90.849	8969.4	52.761	103.90	8693.6	51.139
175.00	92.435	9427.6	53.872	106.56	9219.7	52.684
180.00	94.000	9893.7	54.965	109.18	9759.1	54.217
185.00	95.542	10368	56.041	111.78	10312	55.738
190.00	97.046	10849	57.100	114.35	10877	57.246
195.00	98.527	11338	58.143	116.89	11455	58.743
200.00	99.994	11834	59.171	119.40	12046	60.228
205.00	101.46	12338	60.185	121.89	12649	61.702
210.00	102.92	12849	61.185	124.35	13264	63.164
215.00	104.38	13367	62.173	126.79	13892	64.615
220.00	105.84	13893	63.149	129.20	14532	66.056
225.00	107.30	14426	64.114	131.60	15184	67.486
230.00	108.76	14966	65.068	133.97	15848	68.906
235.00	110.23	15513	66.014	136.33	16524	70.315
240.00	111.70	16068	66.950	138.66	17212	71.715
245.00	113.19	16630	67.879	140.98	17911	73.105
250.00	114.70	17200	68.800	143.28	18621	74.485
255.00	116.24	17777	69.715	145.57	19344	75.857
260.00	117.78	18362	70.625	147.84	20077	77.219
265.00	119.40	18955	71.530	150.10	20822	78.573
270.00	121.07	19557	72.431	152.35	21578	79.919
273.15	122.14	19940	72.999	153.76	22060	80.762
275.00	122.78	20166	73.331	154.59	22345	81.256
280.00	124.53	20784	74.230	156.81	23124	82.585
285.00	126.31	21412	75.128	159.03	23914	83.907
290.00	128.12	22048	76.026	161.25	24714	85.222
295.00	129.95	22693	76.925	163.45	25526	86.529
298.15	131.12	23104	77.491	164.84	26043	87.349
300.00	131.80	23347	77.824	165.65	26349	87.829
305.00	133.67	24011	78.724	167.85	27183	89.123
306.14	134.10	24163	78.929	168.35	27374	89.417
Liquid						
306.14	162.42	42242	137.98	227.40	27374	89.417
310.00	162.46	42869	138.29	229.43	28256	91.148
315.00	162.58	43681	138.67	232.03	29409	93.364
320.00	162.75	44495	139.05	234.60	30576	95.550
325.00	162.94	45309	139.41	237.12	31755	97.709
330.00	163.20	46124	139.77	239.61	32947	99.840

$H_{0}^{c}$ is the enthalpy of the crystal at the saturation pressure at 0 °K.

**Table 14:** Molal thermal functions for methylphosphonyl chlorofluoride (CH_3_POClF) Gram molecular weight = 116.460 g, *T* deg K = *t* deg C + 273.15

*T*	*C*_sat_	$\left(H_{T}-H_{0}^{c}\right)$	$\frac{\left(H_{T}-H_{0}^{c}\right)}{T}$	*S_T_*	$-\left(G_{T}-H_{0}^{c}\right)$	$\frac{-\left(G_{T}-H_{0}^{c}\right)}{T}$
Solid						
*°K*			*J*/*deg*	*J*/*deg*		*J*/*deg*
0.00	0.000	0.000	0.000	0.000	0.000	0.000
5.00	.373	.468	.093	.125	.157	.031
10.00	2.915	7.398	.740	.988	2.485	.248
15.00	8.361	34.672	2.311	3.125	12.209	.814
20.00	14.774	92.574	4.629	6.412	35.676	1.784
25.00	20.448	181.14	7.240	10.341	77.397	3.096
30.00	25.151	295.39	9.846	14.494	139.44	4.648
35.00	29.267	431.69	12.334	18.688	222.41	6.354
40.00	32.731	586.98	14.674	22.830	326.24	8.156
45.00	35.749	758.28	16.851	26.862	450.52	10.012
50.00	38.533	944.08	18.882	30.775	594.67	11.893
55.00	41.109	1143.3	20.786	34.570	758.08	13.783
60.00	43.575	1355.0	22.584	38.253	940.18	15.670
65.00	45.949	1578.9	24.290	41.835	1140.4	17.545
70.00	48.261	1814.4	25.920	45.326	1358.4	19.405
75.00	50.540	2061.4	27.486	48.733	1593.6	21.247
80.00	52.778	2319.7	28.997	52.066	1845.6	23.070
85.00	54.982	2589.1	30.460	55.332	2114.1	24.872
90.00	57.153	2869.5	31.883	58.537	2398.8	26.653
95.00	59.282	3160.6	33.269	61.684	2699.4	28.414
100.00	61.379	3462.3	34.623	64.778	3015.6	30.156
105.00	63.444	3774.3	35.946	67.823	3347.1	31.877
110.00	65.471	4096.6	37.242	70.821	3693.7	33.579
115.00	67.468	4429.0	38.513	73.776	4055.2	35.263
120.00	69.436	4771.3	39.761	76.689	4431.4	36.928
125.00	71.373	5123.3	40.986	79.563	4822.0	38.576
130.00	73.279	5485.0	42.192	82.399	5227.0	40.207
135.00	75.156	5856.1	43.378	85.200	5646.0	41.822
140.00	77.001	6236.5	44.546	87.967	6078.9	43.421
145.00	78.814	6626.0	45.697	90.701	6525.6	45.004
150.00	80.591	7024.5	46.830	93.403	6985.9	46.572
155.00	82.333	7431.9	47.947	96.074	7459.6	48.126
160.00	84.052	7847.8	49.049	98.715	7946.6	49.666
165.00	85.760	8272.4	50.135	101.33	8446.7	51.192
170.00	87.464	8705.4	51.208	103.91	8959.8	52.705
175.00	89.169	9147.0	52.269	106.47	9485.8	54.204
180.00	90.872	9597.1	53.317	109.01	10024	55.691
185.00	92.574	10056	54.355	111.52	10576	57.166
190.00	94.273	10523	55.383	114.01	11140	58.630
195.00	95.970	10998	56.402	116.48	11716	60.081
200.00	97.665	11483	57.413	118.93	12304	61.522
205.00	99.359	11975	58.415	121.37	12905	62.952
210.00	101.05	12476	59.410	123.78	13518	64.372
215.00	102.74	12986	60.398	126.18	14143	65.781
220.00	104.43	13504	61.380	128.56	14780	67.181
225.00	106.11	14030	62.355	130.93	15429	68.571
230.00	107.80	14565	63.325	133.28	16089	69.953
235.00	109.48	15108	64.289	135.61	16761	71.325
240.00	111.16	15659	65.248	137.94	17445	72.688
245.00	112.84	16219	66.202	140.24	18141	74.044
250.00	114.52	16788	67.151	142.54	18848	75.391
250.70	114.75	16868	67.284	142.86	18948	75.578
Liquid						
250.70	149.91	28723	114.57	190.15	18948	75.578
255.00	150.17	29368	115.17	192.70	19771	77.532
260.00	150.48	30120	115.84	195.62	20741	79.775
265.00	150.80	30873	116.50	198.49	21727	81.988
270.00	151.13	31628	117.14	201.31	22726	84.171
273.15	151.35	32104	117.53	203.06	23363	85.532
275.00	151.47	32384	117.76	204.09	23740	86.326
280.00	151.82	33142	118.37	206.82	24767	88.454
285.00	152.18	33902	118.96	209.51	25808	90.554
290.00	152.55	34664	119.53	212.16	26862	92.628
295.00	152.93	35428	120.09	214.77	27929	94.676
298.15	153.17	35910	120.44	216.40	28608	95.953
300.00	153.31	36194	120.64	217.34	29010	96.699
305.00	153.70	36961	121.18	219.88	30103	98.698
310.00	154.10	37731	121.71	222.38	31208	100.67
315.00	154.50	38502	122.23	224.85	32326	102.62
320.00	154.90	39276	122.74	227.29	33457	104.55
325.00	155.31	40052	123.23	229.69	34599	106.46
330.00	155.72	40829	123.72	232.07	35753	108.35
335.00	156.13	41609	124.20	234.41	36919	110.21

$H_{0}^{c}$ is the enthalpy of the crystal at the saturation pressure at 0 °K.

**Table 15:** Comparison of values of entropy In J/deg-mole, °K = °C+273.15°

	Difluoro		Dichloro		Chlorofluoro
	
	*This work*	*Spice et al.*	*This work*	*Spice et al*	*This work*
ΔS, 0–90 °K	51.5	45.2	56.5	50.6	58.5
Δ*S*, 90–298.15 °K	156.8	156.0	108.3	108.6	157.9
*S*, 298.15 °K	208.3	201.2	164.8	159.2	216.4
ΔS fusion, 298.15 °K	……	……	59.9	52.0	……
ΔS vaporization, 298.15 °K.	129.7	131.8	164.1	161.4	155.3
ΔS sublimation, 298.15 °K.	……	……	224.0	213.4	……
ΔS compression to 1 atm, 298.15 °K.	−25.3	−25.1	−49.1	−49.4	−36.7
*S* gas, 1 atm, 298.15 ° K	312.7±3	307.9±8	339.7±3	323.2±21	335.0±3

## Footnotes

3 Figures in brackets indicate the literature references at the end of this paper

4 Since these measurements, the current through the adiabatic shield heaters has been controlled automatically by means of electronic and electro-mechanical equipment.

5 The authors are grateful to Professor J. E. Spice for permission to make these comparisons prior to the publication of his results.
